# The VSG C-terminal domain is inaccessible to antibodies on live trypanosomes

**DOI:** 10.1016/j.molbiopara.2010.11.004

**Published:** 2011-02

**Authors:** Angela Schwede, Nicola Jones, Markus Engstler, Mark Carrington

**Affiliations:** a Department of Biochemistry, Tennis Court Road, Cambridge CB2 1QW, UK; b Department of Cell and Developmental Biology, Theodor-Boveri-Institute, University of Wuerzburg, Am Hubland, 97074 Wuerzburg, Germany

**Keywords:** Trypanosome, VSG, *Trypanosoma brucei*, Cell surface, Antibody

## Abstract

In the mammalian host, the *Trypanosoma brucei* cell surface is covered with a densely packed protein coat of a single protein, the variant surface glycoprotein (VSG). The VSG is believed to shield invariant surface proteins from host antibodies but there is limited information on how far antibodies can penetrate into the VSG monolayer. Here, the VSG surface coat was probed to determine whether it acts as a barrier to binding of antibodies to the membrane proximal VSG C-terminal domain. The binding of C-terminal domain antibodies to VSG221 or VSG118 was compared with antibodies recognising the cognate whole VSGs. The C-terminal VSG domain was inaccessible to antibodies on live cells but not on fixed cells. This provides further evidence that the VSG coat acts as a barrier and protects the cell from antibodies that would otherwise bind to some of the other externally disposed proteins.

## Graphical abstract

**Graphical abstract:**

The VSG coat of *Trypanosoma brucei* prevents access of antibodies to the VSG C-terminal domain.

**Figure:**
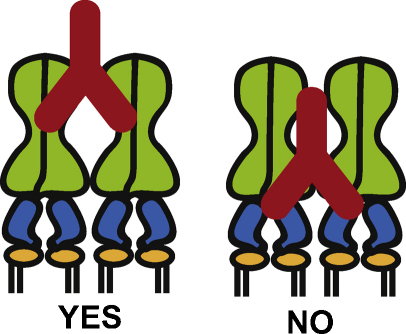

**Research highlights:**

▶ Antisera raised against recombinant VSG C-terminal domains. ▶ Anti-VSG C-terminal domain sera recognise fixed but not live cells. ▶ Support for model where diffusion barrier is at the base of the VSG N-terminal domain.

The variant surface glycoprotein (VSG) is the major cell surface protein of bloodstream forms trypanosomes and forms a protective coat that covers the entire extracellular surface of the cell. Synthesis of VSG coat is required for viability in the bloodstream and tissue fluids of a mammalian host [1]. The VSG coat protects the parasite from the immune system and complement by several mechanisms: (a) VSG shed from the membrane of dying cells modulates the responses of the immune system [2]. (b) The VSG layer protects against complement [3] and protects invariant surface proteins but possibly not entirely through prevention of binding [4,5]. (c) At low antibody titres hydrodynamic flow forces produced by parasite motility drag antibody/VSG complexes to the flagellar pocket of the trypanosomes where they are endocytosed [6] and the antibodies degraded whereas the VSG is recycled back to the surface [7]. These strategies do not provide long-term protection, hosts generate high antibody titres against VSGs and once the titre is high enough antibody-mediated killing occurs that can be reproduced *in vitro* using either complement action or opsonization [8]. However, complete elimination of the population does not normally happen as antigenic variation of the VSG (reviewed in [9]) results in a subset of cells expressing a different VSG and thus escaping recognition for a few more days. Only one *VSG* gene is expressed at any one time and the active *VSG* gene can be changed by transcriptional switching or gene conversion or telomere exchange.

VSGs have two domains: the N-terminal domain is elongated and the core of the domain is formed by a 10 nm coiled coil [10] with the long axis perpendicular to the plane of the plasma membrane. The C-terminal domain is small [11,12] and links the VSG to the membrane via a GPI-anchor on the C-terminal residue [13] (Fig. 1A). Different VSGs are highly divergent at the amino acid level [14] but nevertheless they have very similar structures [10,12]. Measurements of the cell surface area, VSG size, VSG copy number and subcellular localization [15,16] can be combined to show that the VSG is packed at a very high density, close to the possible maximum [17].

The VSG coat is not absolutely uniform as there are other proteins present on the external face of the plasma membrane, for example *ESAG4* and related genes encode a heterogeneous family of type 1 transmembrane proteins localized to the flagellum having an extracellular domain of 70–80 kDa and a cytoplasmic domain encoding an adenylate cyclase [18]. How much such proteins dilute the VSG is unknown as the copy number of the majority has not been determined. An exception is invariant surface glycoproteins (ISGs) that are also a heterogeneous family of type 1 transmembrane proteins localized over the whole cell surface [19–21]. ISGs have a small cytoplasmic domain and an extracellular domain that is a similar size and probably structurally related to VSGs [22]. The copy number for individual ISGs has been estimated to between 5 × 10^4^ and 7 × 10^4^ [19,21] and if this level of expression is extended to the entire ISG family it is likely that there are ∼2 × 10^5^ ISGs in total, equivalent to one ISG for every 50 VSG molecules. Since the ISGs are the most abundant known non-VSG cell surface proteins [19], it is likely that VSG is >95% of the externally disposed protein.

To test the effectiveness of the VSG coat as a barrier, live trypanosomes have been probed with VSG and ISG antibodies and a lectin, concanavalin A (Con A). For VSGs, there are three reports of the partial characterization of epitopes recognised by MoAbs that bind live cells. In the first [23,24], the binding site was localized to a cyanogen bromide peptide that runs along the full length of the longitudinal axis of the VSG and provides limited information how far an immunoglobulin can penetrate. In the second, the epitopes were mapped to the top half of the VSG N-terminal domain [25]. In contrast the third, analysis [26] showed that the MoAb penetrated to the membrane proximal part of the N-terminal domain, at least halfway through the VSG coat.

Con A binds α-linked mannose residues in oligosaccharides. The Con A monomer is a 29 kDa and under normal physiological conditions Con A is in dimer/tetramer equilibrium [27] so the use of Con A is effectively probing the VSG coat with a 58 kDa protein. VSGs have between 0 and 5 N-linked glycosylation sites, the structure of the oligosaccharides at these sites vary [17,28–30], but they will all be bound by Con A. Live trypanosomes expressing most VSGs are not bound by Con A; however, a minority of VSGs allow Con A binding and subsequent agglutination (see [31–33] as examples). These observations can be interpreted as showing the disposition of the N-linked glycan within the VSG coat is variable with some being accessible, and that the 58 kDa Con A cannot penetrate to N-linked glycans proximal to the membrane and especially those attached to the C-terminal domain.

An independent method for probing the VSG coat was to determine whether ISG antibodies bound live cells, the results are variable with binding to both ISG75 and ISG65 has been reported in some cases [4,5]. The observations about ISGs are difficult to fully integrate into a model of the VSG coat as ISG structures and disposition are not known. However, immunization with ISGs does not protect mice [4].

Here, antibodies raised against recombinant C-terminal domains of two VSGs have been used to probe the VSG coat. Two polyclonal antisera were raised against recombinant C-terminal domains from VSGs MITat1.5 (anti-118CTD) and MITat1.2 (anti-221CTD) [11]. Two polyclonal antisera raised against purified native VSGs were used (anti-118 and anti-221) and a murine monoclonal antibody recognising VSG MITat1.6 (anti-121). Western blotting showed that all antibodies were specific (Fig. 1B).

Immunofluorescence analysis showed that all antibodies were able to bind to fixed cells expressing the cognate VSG but not to cells expressing a different VSG (Fig. 2). Under the conditions used, the anti-118 and both anti-221 antibodies produced a low level of signal when used with cells expressing MITat1.6 (VSG121). The origin of this signal is unclear and does not affect the interpretation of the results.

Immunofluorescence experiments with fixed cells were used to titre the different antisera so that the final concentrations used gave similar signal intensities. The same antibody concentrations were then used to determine antibody binding to live cells. Only the antibodies recognising the whole VSG bound to cells expressing the cognate VSG, the signal from live cells was weaker than from fixed cells and a longer acquisition time was used for collecting images. No signal was observed when antibodies recognising the C-terminal VSG domains were used indicating that the C-terminal domain was not accessible to the antibodies.

The antibodies recognising the whole VSGs in this study gave weaker signals on live cells than on fixed cells presumably because the disruption of the surface coat caused by fixation results in greater accessibility. This finding is consistent with the reports that only some VSG monoclonal antibodies are able to bind live cells due to inaccessibility of some epitopes [23,24,26,34–37]. Here, polyclonal antibodies that recognise the VSG C-terminal domain did not bind live cells providing strong evidence that the VSG monolayer is able to exclude antibodies from proteins close to the plasma membrane. These results support the interpretation of earlier experiments that demonstrated that N-linked glycans attached to the C-terminal domain were inaccessible to Con A. The VSG coat is approximately 15 nm thick, recent structural studies on the VSG C-terminal domain [11] indicate that it lies within 5 nm of the plasma membrane. Overall, these and earlier data support a model where much of the N-terminal domain of the VSG is accessible to immunoglobulins whereas the C-terminal domain is not. The accessibility barrier is probably close to the membrane proximal base of the VSG N-terminal domain, this location is the point of greatest cross-sectional area of the VSG. The next question to be answered is how small does a molecule have to be in order to access the C-terminal domain and below?

## Figures and Tables

**Fig. 1:**
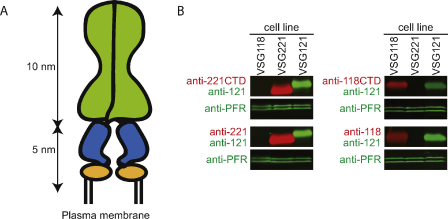
(A) Schematic representation of a VSG. The N-terminal domain is depicted in green, the C-terminal domain in blue and the GPI-anchor in yellow. (B) Western blotting to test antibody specificity. Cell lines expressing different VSGs were used to prepare total cell lysates: MITat1.5 (VSG118); MITat1.2 (VSG221) and MITat1.6 (VSG121). To prepare samples for Western blotting, cells were washed in HMI-9 without serum, the cell were resuspended at 3 × 10^8^/ml and 0.5 volumes of 3× SDS-PAGE sample buffer added and the sample was incubated at 100 °C immediately. Using this procedure, there was no detectable activation of the glycosylphosphatidylinositol-specific phospholipase C and production of the cross-reacting determinant. The polyclonal rabbit antibodies recognising the C-terminal VSG domains (anti-118CTD and anti-221CTD) and the antibodies recognising the whole VSGs (anti-118 and anti-221) were detected with Alexa680-conjugated goat anti-rabbit as a red signal. As a control, a monoclonal mouse anti-MITat1.6 (anti-121) was used and detected with infrared dye 800-conjugated goat anti-mouse as a green signal. An anti-PFR1 monoclonal was used as a loading control and was also detected as a green signal. The anti-118CTD was raised in rabbit against residues 328–429 (the C-terminus) expressed in *E. coli* and purified as described for the C-terminal di-domain of VSG ILTat 1.24 [12]. Anti-221CTD was raised against residues 359–433 (the C-terminus) expressed and purified as described [11]. The anti-221 was produced in the same way to that previously described [37].

**Fig. 2:**
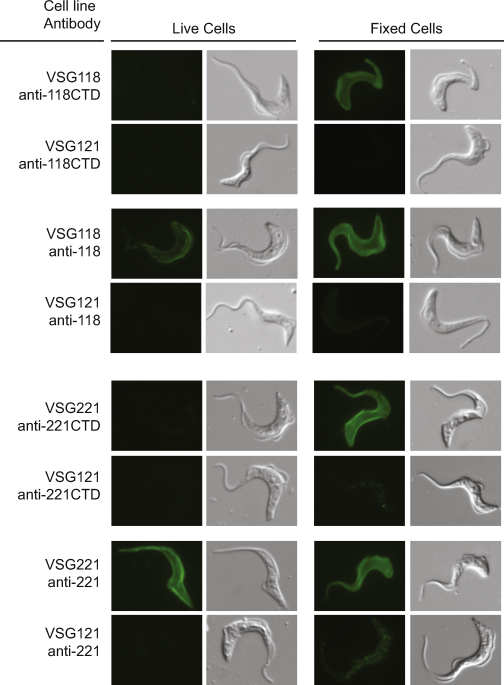
Antibodies recognising VSG C-terminal domains are not able to bind to live cells but do recognise the cells after fixation whereas antibodies recognising the whole VSGs bound both live and fixed cells. Cells were washed in HMI-9 without serum and resuspended in HMI-9 without serum prior to fixation by the addition of an equal volume of 8% paraformaldehyde in phosphate buffered saline with 45.9 mM sucrose and 10 mM glucose and incubation at 0 °C for 15 min and then another 15 min after transfer to room temperature. In experiments to determine binding to live trypanosomes, cells were first cooled to 0 °C for 20 min, incubated with antibodies for 10 min, centrifuged and resuspended in ice cold HMI-9 without serum and then fixed by the addition of an equal volume of 8% paraformaldehyde in phosphate buffered saline with 45.9 mM sucrose and 10 mM glucose and incubated at 0 °C for 15 min and then another 15 min after transfer to room temperature. After fixation standard immunofluorescence techniques were used. The VSG121 cell line served as a control for non-specific signal.

## References

[1] Sheader K., Vaughan S., Minchin J., Hughes K., Gull K., Rudenko G. (2005). Variant surface glycoprotein RNA interference triggers a precytokinesis cell cycle arrest in African trypanosomes. Proc Natl Acad Sci USA.

[2] Mansfield J.M., Paulnock D.M. (2005). Regulation of innate and acquired immunity in African trypanosomiasis. Parasite Immunol.

[3] Ferrante A., Allison A.C. (1983). Alternative pathway activation of complement by African trypanosomes lacking a glycoprotein coat. Parasite Immunol.

[4] Ziegelbauer K., Overath P. (1993). Organization of two invariant surface glycoproteins in the surface coat of *Trypanosoma brucei*. Infect Immun.

[5] Chung W.L., Carrington M., Field M.C. (2004). Cytoplasmic targeting signals in transmembrane invariant surface glycoproteins of trypanosomes. J Biol Chem.

[6] Engstler M., Pfohl T., Herminghaus S. (2007). Hydrodynamic flow-mediated protein sorting on the cell surface of trypanosomes. Cell.

[7] Pal A., Hall B.S., Jeffries T.R., Field M.C. (2003). Rab5 and Rab11 mediate transferrin and anti-variant surface glycoprotein antibody recycling in *Trypanosoma brucei*. Biochem J.

[8] McLintock L.M., Turner C.M., Vickerman K. (1993). Comparison of the effects of immune killing mechanisms on *Trypanosoma brucei* parasites of slender and stumpy morphology. Parasite Immunol.

[9] Schwede A., Carrington M. (2010). Bloodstream form trypanosome plasma membrane proteins: antigenic variation and invariant antigens. Parasitology.

[10] Blum M.L., Down J.A., Gurnett A.M., Carrington M., Turner M.J., Wiley D.C. (1993). A structural motif in the variant surface glycoproteins of *Trypanosoma brucei*. Nature.

[11] Chattopadhyay A., Jones N., Nietlispach D. (2005). Structure of the C-terminal domain from *Trypanosoma brucei* variant surface glycoprotein MITat1.2. J Biol Chem.

[12] Jones N.G., Nietlispach D., Sharma R. (2008). Structure of a glycosylphosphatidylinositol-anchored domain from a trypanosome variant surface glycoprotein. J Biol Chem.

[13] Ferguson M.A.J., Homans S.W., Dwek R.A., Rademacher T.W. (1988). Glycosylphosphatidylinositol moiety that anchors *Trypanosoma brucei* variant surface glycoprotein to the membrane. Science.

[14] Carrington M., Miller N., Blum M., Roditi I., Wiley D., Turner M. (1991). Variant specific glycoprotein of *Trypanosoma brucei* consists of two domains each having an independently conserved pattern of cysteine residues. J Mol Biol.

[15] Jackson D.G., Owen M.J., Voorheis H.P. (1985). A new method for the rapid purification of both the membrane-bound and released forms of the variant surface glycoprotein from *Trypanosoma brucei*. Biochem J.

[16] Grunfelder C.G., Engstler M., Weise F. (2003). Endocytosis of a glycosylphosphatidylinositol-anchored protein via clathrin-coated vesicles, sorting by default in endosomes, and exocytosis via RAB11-positive carriers. Mol Biol Cell.

[17] Mehlert A., Bond C.S., Ferguson M.A. (2002). The glycoforms of a *Trypanosoma brucei* variant surface glycoprotein and molecular modeling of a glycosylated surface coat. Glycobiology.

[18] Paindavoine P., Rolin S., Van Assel S. (1992). A gene from the variant surface glycoprotein expression site encodes one of several transmembrane adenylate cyclases located on the flagellum of *Trypanosoma brucei*. Mol Cell Biol.

[19] Ziegelbauer K., Overath P. (1992). Identification of invariant surface glycoproteins in the bloodstream stage of *Trypanosoma brucei*. J Biol Chem.

[20] Ziegelbauer K., Multhaup G., Overath P. (1992). Molecular characterization of two invariant surface glycoproteins specific for the bloodstream stage of *Trypanosoma brucei*. J Biol Chem.

[21] Jackson D.G., Windle H.J., Voorheis H.P. (1993). The identification, purification, and characterization of two invariant surface glycoproteins located beneath the surface coat barrier of bloodstream forms of *Trypanosoma brucei*. J Biol Chem.

[22] Carrington M., Boothroyd J. (1996). Implications of conserved structural motifs in disparate trypanosome surface proteins. Mol Biochem Parasitol.

[23] Miller E.N., Allan L.M., Turner M.J. (1984). Mapping of antigenic determinants within peptides of a variant surface glycoprotein of *Trypanosoma brucei*. Mol Biochem Parasitol.

[24] Miller E.N., Allan L.M., Turner M.J. (1984). Topological analysis of antigenic determinants on a variant surface glycoprotein of *Trypanosoma brucei*. Mol Biochem Parasitol.

[25] Baltz T., Giroud C., Bringaud F., Eisen H., Jacquemot C., Roth C.W. (1991). Exposed epitopes on a *Trypanosoma equiperdum* variant surface glycoprotein altered by point mutations. EMBO J.

[26] Hsia R., Beals T., Boothroyd J.C. (1996). Use of chimeric recombinant polypeptides to analyse conformational, surface epitopes on trypanosome variant surface glycoproteins. Mol Microbiol.

[27] Senear D.F., Teller D.C. (1981). Thermodynamics of concanavalin A dimer-tetramer self-association: sedimentation equilibrium studies. Biochemistry.

[28] Zamze S.E., Ashford D.A., Wooten E.W., Rademacher T.W., Dwek R.A. (1991). Structural characterization of the asparagine-linked oligosaccharides from *Trypanosoma brucei* type II and type III variant surface glycoproteins. J Biol Chem.

[29] Zamze S.E., Wooten E.W., Ashford D.A., Ferguson M.A., Dwek R.A., Rademacher T.W. (1990). Characterisation of the asparagine-linked oligosaccharides from *Trypanosoma brucei* type-I variant surface glycoproteins. Eur J Biochem.

[30] Mehlert A., Sullivan L., Ferguson M.A. (2010). Glycotyping of *Trypanosoma brucei* variant surface glycoprotein MITat1.8. Mol Biochem Parasitol.

[31] Cross G.A.M., Johnson J.G., Van den Bosche H. (1976). Structure and organisation of the variant-specific surface antigens of *Trypanosoma brucei*. Biochemistry of parasite and host–parasite relationships.

[32] Baltz T., Baltz D., Pautrizel R. (1976). Affinite de la Concanavaline A pour *Trypanosoma equiperdum* applications a l’isolement de la fraction glycoproteique specifique du type antigenique. Ann Immunol (Inst Pasteur).

[33] Balber A.E., Frommel T.O. (1988). *Trypanosoma brucei gambiense* and *T. b. rhodesiense*: concanavalin A binding to the membrane and flagellar pocket of bloodstream and procyclic forms. J Protozool.

[34] Barbet A.F., Davis W.C., McGuire T.C. (1982). Cross-neutralization of two different trypanosome populations derived from a single organism. Nature.

[35] Hall T., Esser K. (1984). Topologic mapping of protective and nonprotective epitopes on the variant surface glycoprotein of the WRATat 1 clone of *Trypanosoma brucei rhodesiense*. J Immunol.

[36] Pinder M., van Melick A., Vernet G. (1987). Analysis of protective epitopes on the variant surface glycoprotein of a *Trypanosoma brucei brucei* (DiTat 1.3.) using monoclonal antibodies. Parasite Immunol.

[37] Masterson W.J., Taylor D., Turner M.J. (1988). Topologic analysis of the epitopes of a variant surface glycoprotein of *Trypanosoma brucei*. J Immunol.

